# Anti-diarrheal and anti-inflammatory activities of aqueous extract of the aerial part of *Rubia cordifolia*

**DOI:** 10.1186/s12906-016-1527-9

**Published:** 2017-01-05

**Authors:** Xue-Peng Gong, Yuan-Yuan Sun, Wei Chen, Xia Guo, Jian-Kun Guan, Dong-Yan Li, Guang Du

**Affiliations:** Department of Pharmacy, Tongji Hospital, Tongji Medical College, Huazhong University of Science and Technology, 1095# Hangkong Road, Wuhan, 430030 People’s Republic of China

**Keywords:** Anti-diarrheal, Anti-inflammatory, *Rubia cordifolia* Aerial part, Aqueous extract, Rodent model

## Abstract

**Background:**

In Shaanxi province, China, the aqueous extract of *Rubia cordifolia’s* aerial part (AERCAP) is traditionally used to manage diarrhea. However, there is no scientific evidence to verify the safety and efficacy of its use. The aim of this study was to investigate the anti-diarrheal and anti-inflammatory effects of AERCAP by using a rodent model.

**Methods:**

The anti-diarrheal effects were studied by senna leaf-induced diarrheal and intestinal transit experiments in mice. The anti-inflammatory activity was investigated by trinitrobenzenesulfonic acid (TNBS)-induced colonic inflammation in rats.

**Results:**

The results indicated that AERCAP delayed the onset of semi-solid feces, reduced the evacuation index (EI) in senna leaf-induced diarrheal in mice, and inhibited the propulsive movement in castor oil-induced intestinal transit but not in the normal intestinal transit test. The results were compared with the standard anti-diarrheal drug loperamide. Additionally, oral treatment with AERCAP significantly decreased the macroscopic damage area, improved the microscopic structure, and reduced the malondialdehyde (MDA) content, IL-1β and TNF-α levels in colonic tissue compared with the TNBS control group in rats.

**Conclusions:**

AERCAP exhibited anti-diarrheal and anti-inflammatory activities in a rodent model. The study validated the traditional use of the plant in Chinese herbal medicine as a valuable natural remedy for the treatment of diarrhea.

## Background

Diarrhea is defined as a disorder that is characterized by the discharge of semi-solid or watery fecal matter from the bowel three or more times in a day [1]. Diarrhea is one of the leading causes of morbidity and mortality in developing countries, especially for children under the age of 5 [2]. Although some effective drugs are available around the world, searching for an anti-diarrheal traditional herbal medicine is still encouraged by the World Health Organization (WHO) because of its safety and availability [3].

*Rubia cordifolia* is an important medicinal plant that has numerous ethno-medicinal applications in many indigenous medical systems. In the Indian Ayurvedic system, *Rubia cordifolia* is used to manage dropsy, paralysis, amenorrhea and jaundice [4]. In Chinese Medicine, the root of the *Rubia cordifolia* can stop bleeding, promote blood circulation to remove blood stasis [5] and is commonly used to treat arthritis, hematorrhea, hemostasis and dysmenorrhea [6]. The aerial part of the *Rubia cordifolia*, which is locally named ‘*Guo Shan Long*’ in Shaanxi Province in China, has been used as anti-diarrheal remedy for over a century. The water decoction of the plant can be administered orally to treat diarrhea or used externally via a foot bath when children are too young to take the decoction. The plant is also an ingredient of a formula called ‘Er-Xie-Ting granule’ that is used to treat acute infantile diarrhea [7]. Additionally, the anti-diarrheal effect of this plant has been documented in Chinese Materia Madica Mongolia Volume [8].

Despite the traditional use of this plant, some studies have reported for its anti-oxidative [9], antibacterial [10], anticonvulsant [11], and anti-inflammatory [12] activities. Since the oxidative stress, enteric pathogen infection, deranged intestinal motility are the established etiopathology of diarrhea [13], the studies cited above provide a basis of our experiments which aim to evaluate the safety and pharmacological mechanism of the plant in the treatment of diarrhea. Furthermore, we evaluated the protective effects of AERCAP on TNBS-induced colitis in rats because inflammation is also an established etiopathology of diarrhea.

## Methods

### Plant material

The aerial part of the *Rubia cordifolia* was collected from Hanzhong district, Shaanxi province, China, from August to September 2014. The plant was authenticated by Prof. Yong-Hui Zhang, a taxonomist in the Pharmacy College, Tongji Medical College, Huazhong University of Science and Technology. The plant specimen was deposited at the same college, and the voucher number was NP2014100703.

### Preparation of plant extract

The plant (2 kg) was air dried at room temperature and cut using an electric shredder. The powder was macerated in distilled water (1:10 w/v) for 0.5 h and then the components were extracted twice for 0.5 h each time. The combined aqueous extract solution was filtered and concentrated to dryness under reduced pressure at 65 °C in a rotary evaporator. Finally, it was freeze-dried to obtain 238.9 g dark brown crude powder with 11.95% yield. The aqueous extract was weighed and dissolved in distilled water for use on each day of the experiment.

### Preliminary phytochemical screening test

Phytochemical screening of the AERCAP was performed qualitatively according to the standard methods of coloring and precipitation. The chromatography and spectroscopic methods were used to isolate pure compounds and identify the planar structures of the compounds [14].

### Animals

Male Swiss albino mice (18–20 g) and Wister rats (180–200 g) were obtained from the laboratory animal center of Tongji Hospital, Tongji Medical College, Huazhong University of Science and Technology (license number: SYXK2014-0049). The animals were housed in plastic cages under standard conditions, including a 12-h dark-light cycle and 22 ± 2 °C. Animals had free access to pellet food and water. All animals were acclimatized to laboratory conditions for 1 week and were fasted overnight before the experiments. All study protocols were approved by the Animal Care and Use Committee of Tongji Mediclal College.

### Acute oral toxicity test

The LD_50_ study followed Karber’s method with slight modification [15]. Briefly, 1, 2, 4, or 8 g/kg body weight (8.37 g crude drug/g) of AERCAP was administered by gavage to groups of mice (10 mice per group). The healthy control group was treated with distilled water (40 ml/kg). The mice were then allowed free access to food and water. General behavior changes and mortality were strictly observed during the next 4 h. Such observation was continued for 14 days for any signs of toxicity. Then, a single and maximum dose test was used to evaluate the safety of the extract [15]. The suspension of maximum concentration (13.2 g/kg body weight crude powder) that could pass through the 16th IG pin was prepared and orally administered to each mouse with the maximum filling volume of stomach (0.4 ml/10 g) once daily, and the mice were observed for 14 days as mentioned above.

### Senna leaf-induced diarrheal

The senna leaf decoction was fed to mice to induce diarrhea as previously described by Zhang J. [16] and Tadesse WT. [17] with some modifications. Briefly, the mice were screened by giving 0.4 ml of the senna leaf decoction (2 g crude drug/ml), and only those mice exhibiting diarrhea were selected for the final experiment. Before the commencement of the experiment, 50 mice were randomly allocated into five groups, with 10 mice per group. The negative control group was treated with distilled water (20 ml/kg), and the positive control group was treated with loperamide (4 mg/kg). The test groups were administered 500, 1,000, or 2,000 mg/kg AERCAP. Thirty minutes later, 0.4 ml senna leaf decoction was administered orally to each mouse. Then, each mouse was housed in an individual cage with the floor lined with blotting paper. Wire nets were set into cages 3 cm from the bottom to separate the mice from the blotting paper. Thereafter, animals were observed for 4 h for the following parameters: time to initial semi-solid feces, number of solid, semi-solid and liquid feces. A numerical score was assigned as follows: solid feces = 1, semi-solid feces = 2, and liquid feces = 3. The evacuation index (EI) in the formula below was used to evaluate the severity of diarrhea.

$$ \mathrm{E}\mathrm{I}=\mathrm{solid}\ \mathrm{feces}\times 1+\mathrm{semi}-\mathrm{solid}\ \mathrm{feces}\times 2+\mathrm{liquid}\ \mathrm{feces}\times 3 $$

### Normal intestinal transit

The methods described by de Sales I.R. [18] and Awe E.O. [19] were followed with slight modification. Fifty mice were randomly divided into five groups (*n* = 10). The negative group was given distilled water (20 ml/kg), and the positive group was given loperamide (4 mg/kg). The other groups were administered AERCAP 500, 1,000, or 2,000 mg/kg AERCAP. After 0.5 h, all animals received 0.2 ml red Chinese ink (mainly contain cinnabar). Another 20 min later, all animals were euthanized by cervical dislocation, and the small intestines were removed. The distance traveled by the red Chinese ink from the pylorus to the caecum and the total length of the small intestine were recorded. The intestinal transit percentage was determined using the formula below.

$$ \%\ \mathrm{transit}=\frac{\mathrm{distance}\ \mathrm{traveled}\ \mathrm{b}\mathrm{y}\ \mathrm{red}\ \mathrm{Chinese}\ \mathrm{ink}}{\mathrm{total}\ \mathrm{lenth}\ \mathrm{of}\ \mathrm{the}\ \mathrm{small}\ \mathrm{intestine}} \times 100\% $$

### Castor oil induced intestinal transit

All procedures and protocols described above were repeated, except castor oil (0.2 ml/animal) was administered to each animal 30 min before the administration of red Chinese ink. The intestinal transit percentage was also determined to evaluate the inhibitory action of AERCAP on castor oil-induced intestinal transit in mice.

### Anti-inflammatory activity

To evaluate the anti-inflammatory activity of AERCAP, the acute intestinal inflammation model was established according to Pawar P. and de Almeida A. B. [20, 21]. Rats were randomly divided into 6 groups (*n* = 8). The healthy group and the TNBS group were provided with distilled water, and the other four groups of rats were administered with dexamethasone (0.3 mg/kg) and different doses of AERCAP (250, 500, 1,000 mg/kg) for 5 days before the induction of colitis. On the sixth day, rats were anesthetized with urethane, and the medical infusion tubes were inserted into the anus and advanced 8 cm proximally. TNBS dissolved in 50% ethanol was administered through the tube (4.8 mg in 0.3 ml), whereas the same volume of distilled water was administered to rats in the healthy group. Then, animals were kept in a head-down position for 1 min to prevent solution leakage.

Seventy-two hours later, rats were sacrificed using an overdose of anesthetic. The distal 8 cm of the colon of each rat was removed and opened longitudinally. The luminal content was washed out by saline. Photographs of colon samples were taken using a digital camera to assess the macroscopic lesion. Part of the inflamed tissue sample taken from the distal colon of each animal was fixed in 4% buffered formaldehyde and then embedded in paraffin. Slices were made by a cryostat and then the samples were stained with hematoxylin and eosin for the histological evaluation of colonic damage. The remaining colon tissues were used to generate homogenates for the further assessment of the biochemical parameters.

The anti-oxidative biochemical parameters MDA and myeloperoxidase (MPO) were determined using a commercial biochemical assay kit (Nanjing Jiancheng Bioengineering Institute, Nanjing, China), following the chromometry principle. The pro-inflammatory cytokines TNF-α and IL-1β were quantitatively measured using the enzyme-linked immunosorbent assay (ELISA) kit (R&D Systems China Co. Ltd., Shanghai, China).

### Statistical analysis

The parametric data were expressed as the mean ± standard error of the mean (S.E.M.), and the differences between groups were determined by one-way analysis of variance (ANOVA) followed by Dunnett’s test or LSD test. The non-parametric data were expressed by lower quartile (QL), upper quartile (QU) and median and were analyzed by the Kruskal-Wallis test followed by Dunn’s test. *P* < 0.05 was considered statistically significant. Statistical analysis was performed using the Prism 5.0 software (GraphPad, Inc.).

## Results

### Preliminary phytochemical screening

The preliminary phytochemical analysis revealed the presence of anthraquinones, phenolic acids, tannins and amino acids in AERCAP.

### Acute toxicity

In the LD_50_ study, orally administered graded doses of AERCAP did not produce any mortality or signs of toxicity in the observation period. In the single and maximum dose test, up to 13.2 g/kg body weight (8.37 g crude drug/g) of the orally administered AERCAP did not result in any mortality or behavioral and physical changes during the observation period.

### Senna leaf-induced diarrhea

**Fig. 1:**
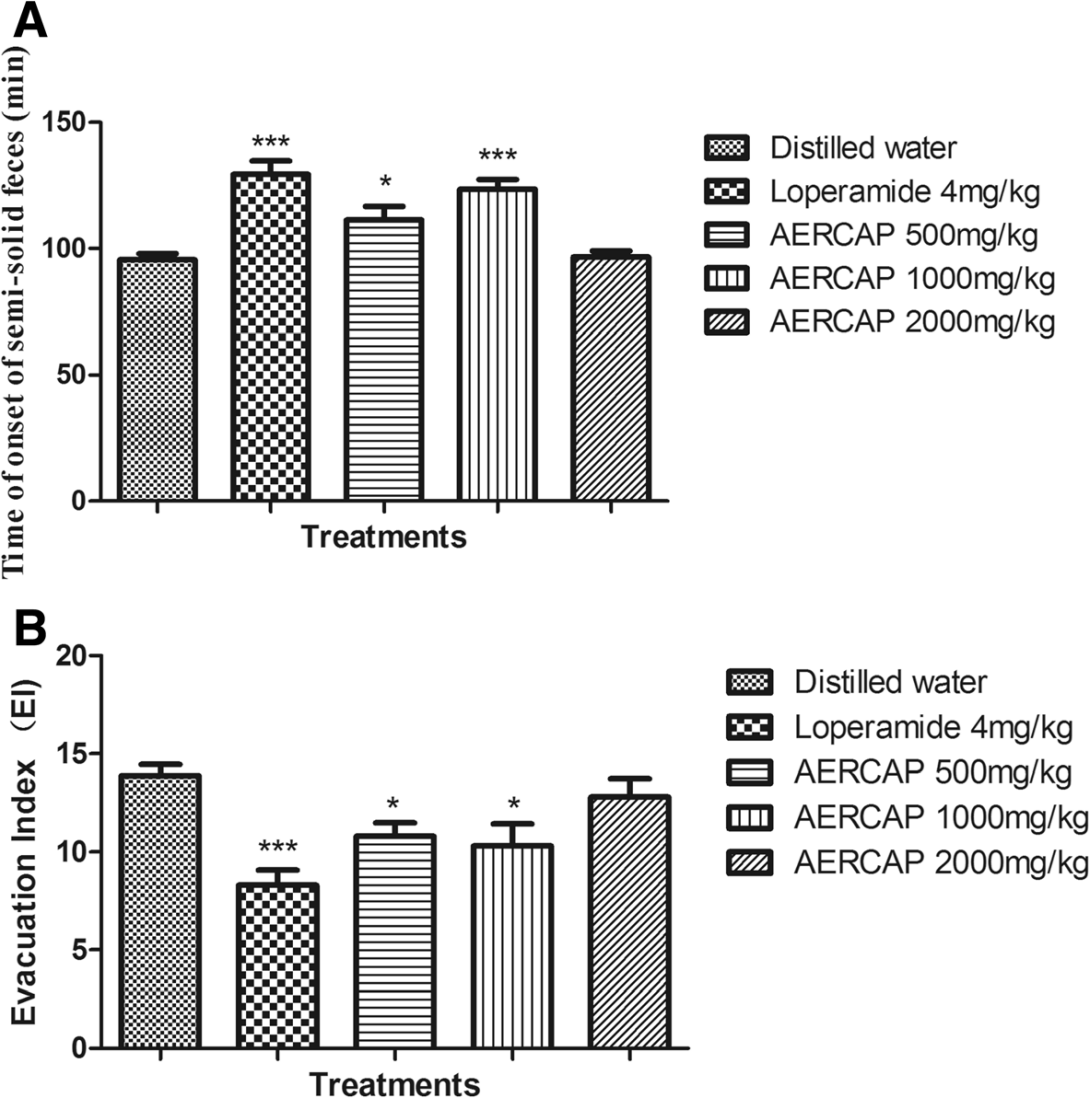
Effect of AERCAP on senna leaf-induced diarrhea in mice. **a** Time of onset of semi-solid feces (min), ANOVA:F_(4,45)_ = 13.98, *p* < 0.001, *n* = 10; **b** Evacuation index (EI), ANOVA:F_(4,45)_ = 6.67, *p* < 0.001, *n* = 10; followed by Dunnett’s test, *p < 0.05, ***p < 0.001, compared with the negative control group; volumes presented as the mean ± S.E.M

In the 4 h after senna leaf decoction administration, all of the mice in the negative control group produced liquid feces, with a semi-solid feces onset time of 95.5 ± 2.5 min. In addition, some of the mice in other groups did not produce liquid feces. The AERCAP 500 mg/kg and 1,000 mg/kg AERCAP groups significantly inhibited diarrhea by increasing the onset time of semi-solid feces and decreasing the EI score compared with the negative control group (*p* < 0.05–0.001). Such inhibitory effects were not noted in the 2,000 mg/kg AERCAP group (Fig. 1a, b). In addition, the standard anti-diarrheal drug loperamide produced significantly inhibitory effects on those two parameters (*p* < 0.001).

### Normal intestinal transit

**Fig. 2:**
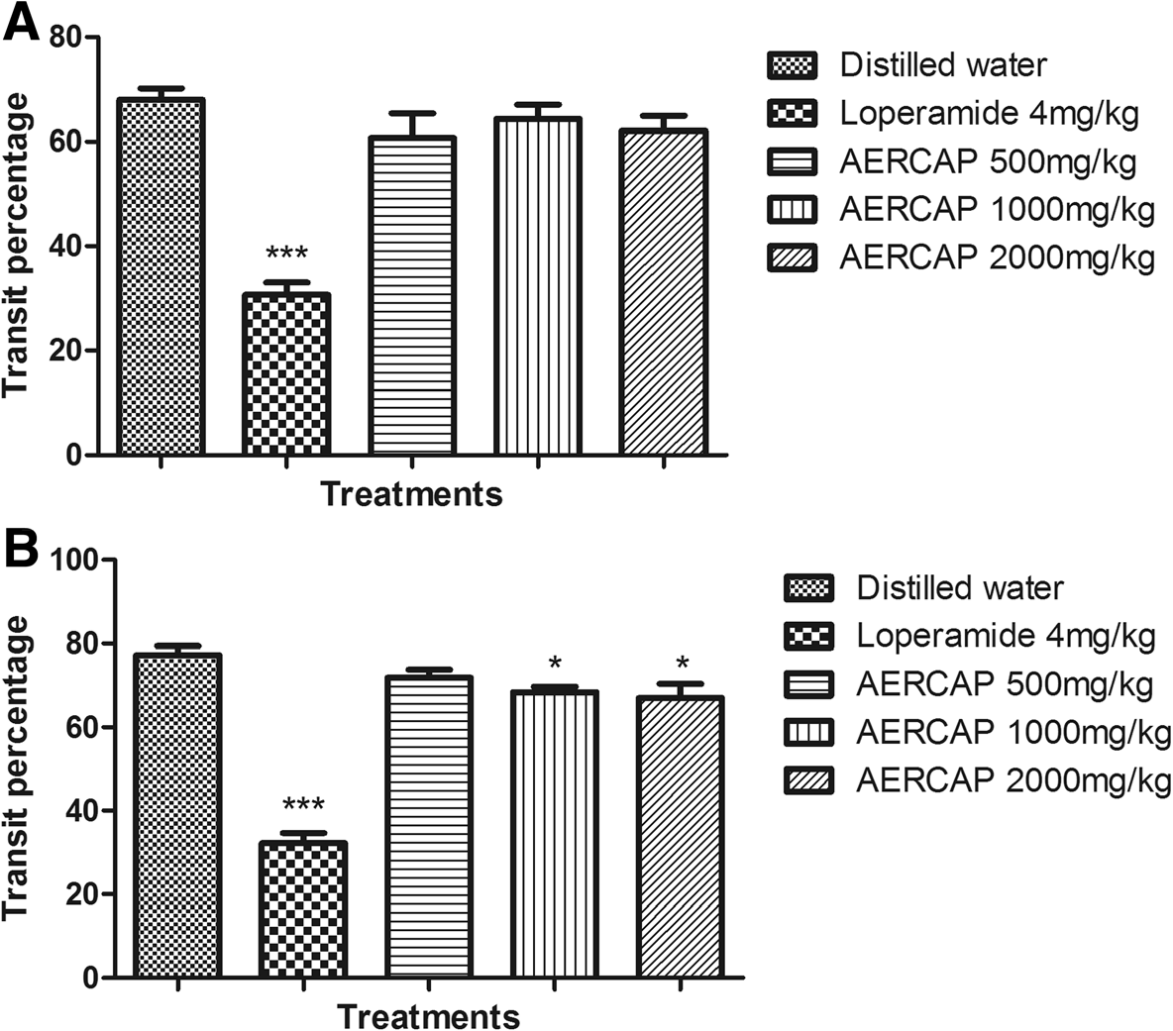
Effect of AERCAP on intestinal transit in mice. **a** Normal intestinal transit, ANOVA, F_(4,45)_ = 23.67, *p* < 0.001, *n* = 10; **b** Castor oil stimulated intestinal transit, ANOVA, F_(4,45)_ =57.13, *p* < 0.001, *n* = 10; followed by Dunnett’s test, * *p* < 0.05, *** *p* < 0.001, compared to the negative control group; volumes presented as the mean ± S.E.M

The effect of AERCAP on the normal intestinal transit is depicted in Fig. 2a. Graded doses of the extract did not exhibit any inhibition effect on the normal intestinal transit compared with the negative control. However, the loperamide group demonstrated a significant inhibitory effect in normal intestinal transit (*p* < 0.001).

### Castor oil-induced intestinal transit

As shown in Fig. 2b, 20 min after the intra-gastric administration of red Chinese ink, the ink traveled 77.32% ± 2.18 of total length of the small intestine in the negative control group. Such transit movements were inhibited in every AERCAP group, but only the 1,000 mg/kg and 2,000 mg/kg groups exhibited significant differences (*p* < 0.05). In the loperamide group, the propulsive movement was significantly inhibited compared with all AERCAP doses (*p* < 0.001).

### Anti-inflammatory activities

**Fig. 3:**
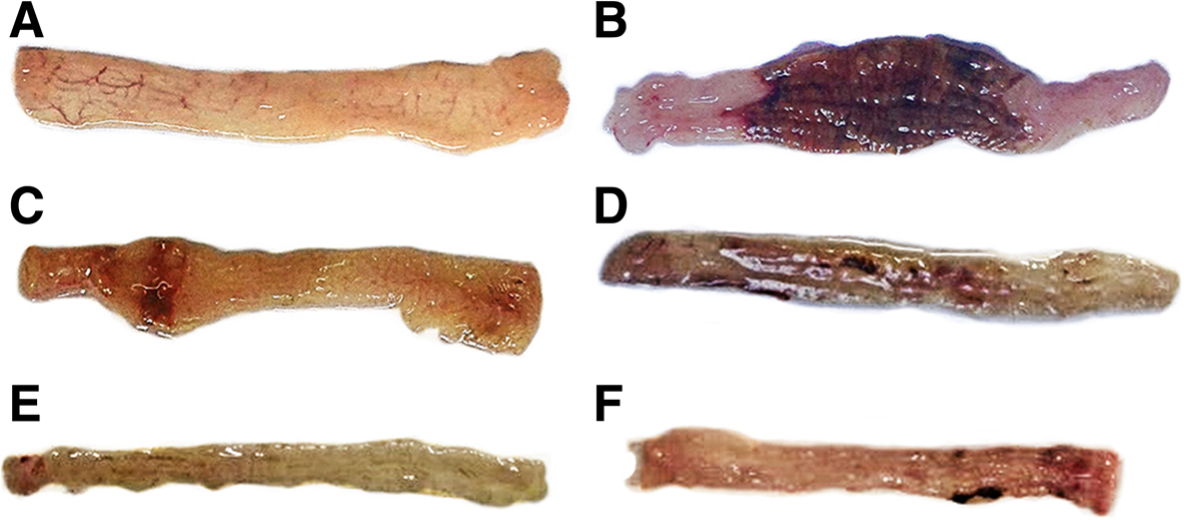
Macroscopic images of the colon mucosa. **a** healthy control group, **b** TNBS control group, **c** dexamethasone group, **d** 250 mg/kg AERCAP group, **e** 500 mg/kg AERCAP group, **f** 1,000 mg/kg AERCAP group

After treatment with TNBS, rats exhibited hypomotility, anorexia, diarrhea and prostration. The macroscopic inspection of the colon showed that the TNBS control group presented the most severe mucosal damage with edema, deep ulceration and necrosis (Fig. 3b). On the contrary, in the dexamethasone- and AERCAP-treated groups, we observed a significant reduction in the lesion area in the colon mucosal, as shown in Fig. 3c, d, e, f.

**Fig. 4:**
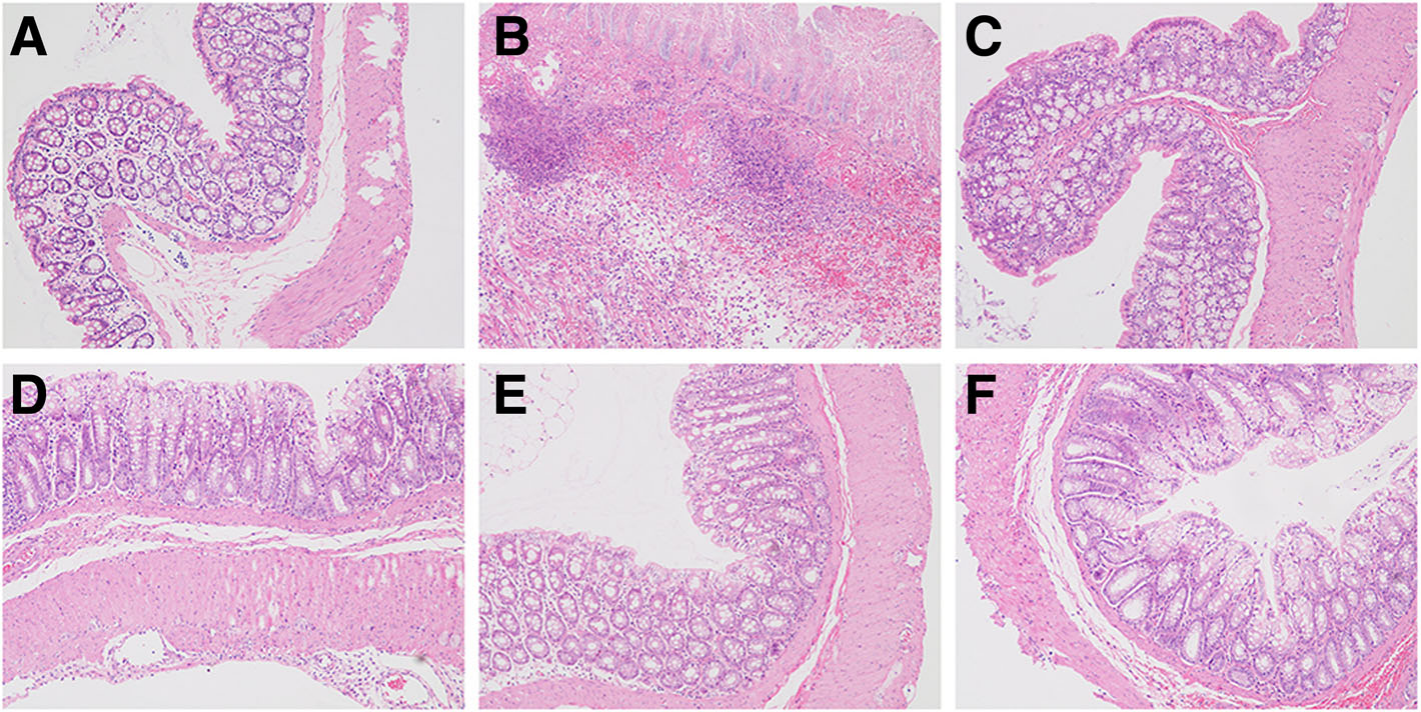
Histological features of colon segments stained with hematoxylin and eosin, **a** healthy control group, **b** TNBS control group, **c** dexamethasone group, **d** AERCAP 250 mg/kg group, **e** AERCAP 500 mg/kg group, **f** AERCAP 1,000 mg/kg group

The histological analysis of the tissue from the healthy control group showed a typical normal structure of epithelial cell layer, lamina propria and sub-mucosa (Fig. 4a). The TNBS control group exhibited severe disorganization with transmural necrosis, edema, epithelial cell disruption and diffused inflammatory cell infiltration in the mucosa and sub-mucosa (Fig. 4b). In the 250 and 1,000 mg/kg AERCAP groups, the mucosal crypts exhibited slight distortion, and the inflammatory cell infiltration and edema of the sub-mucosa were present but were less damaged than those observed in the TNBS control group (Fig. 4d, f). The positive control group and the 500 mg/kg AERCAP group exhibited integral epithelial mucosa and normal mucosal crypts, similar to the healthy control group.

**Fig. 5:**
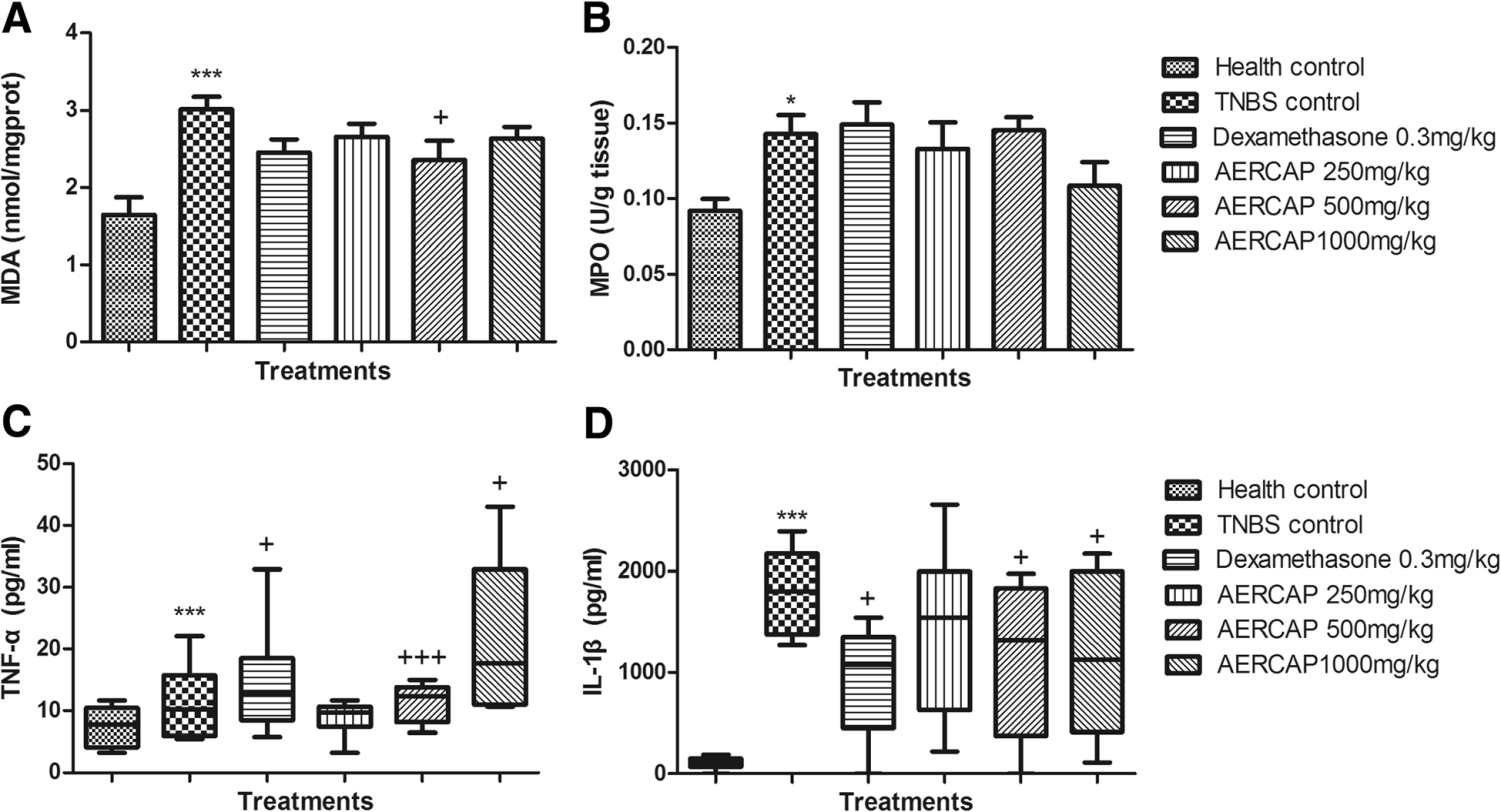
Anti-oxidative and anti-inflammatory effects of AERCAP on TNBS-induced colitis in rats. **a**. MDA content, ANOVA, F_(5,38)_ = 5.88, *p* < 0.001; **b** MPO activity, ANOVA, F_(5,38)_ = 0.290, *p* < 0.05; *n* = 6–8, followed by LSD test; columns present the mean ± S.E.M. **c** TNF-α level, **d** IL-1β level, Kruskal-Wallis test, *p* < 0.01, followed by Dunn’s multiple comparison test, *n* = 6–8, boxes presented Q25%-Q75%. *p < 0.05, ***p < 0.001, compared with health control group; +*p* < 0.05, +++*p* < 0.001, compared with the TNBS control group

The biochemical assay showed that the reaction between MDA and thiobarbituric acid (TBA) yielded a pink red product, which has a maximum absorption peak at 532 nm. The MDA content increased significantly in the TNBS control group compared with the healthy group (*p* < 0.001). The dexamethasone and AERCAP groups decreased the MDA content to a certain extent, but only the 500 mg/kg AERCAP group exhibited a significant difference (*p* < 0.05). Adding O-dianisidinedihydrochloride to the MPO-hydrogen peroxide (H_2_O_2_) complex in the homogenate can produce a yellow compound that has a maximum absorption peak at 460 nm. The MPO activity increased significantly in TNBS control group compared with the healthy group. No difference was observed between any other treated groups and the TNBS control group. In the ELISA detection, our results also showed that the TNF-α and IL-1β levels were significantly increased in the TNBS control group compared with the healthy control group (*p* < 0.001). Dexamethasone and AERCAP treatment significantly reduced the levels of these two inflammatory cytokines compared with the TNBS control group (*p* < 0.05–0.001) (Fig. 5).

## Discussion

In the present study, the crude aqueous extract was used for the experiments because it represents the closest form of the traditional product. The oral administration of AERCAP up to 13.2 g/kg did not produce any mortality or toxic effects. According to Lorke [22], any substance that is not toxic at 5 g/kg can be considered relatively safe. These results demonstrate the safety profile of the AERCAP and are useful for understanding the following in vivo pharmacological studies.

Although the etiology of diarrhea is complicated, there are some established mechanisms that can elicit the disease, such as increased electrolyte secretion (secretory diarrheal), infectious or inflammatory induced mucosal injury (exudalive diarrhea), and deranged intestinal motility. Various effective laxatives, such as castor oil and senna leaf, are used to establish animal models of diarrhea. In our study, we used senna leaf to create a diarrhea model. Senna leaf is one of the most common herbal drugs that are used against constipation or as effective laxatives to evacuate the bowel prior to diagnostic radiographs in China [23]. The induction of diarrhea by senna leaf is attributed to its active ingredient sennoside A [24]. This active component can stimulate the synthesis of prostaglandin, histamine and serotonin and can inhibit Na^+^ and K^+^ - ATPase to alter fluid transport across the intestinal mucosa [25].

In our study, senna leaf produced intense diarrhea in mice. The administration of 500 mg and 1,000 mg/kg AERCAP significantly relieved symptoms by prolonging the onset time of wet feces and reducing the frequency of wet feces. However, such effects were not noted with 2,000 mg/kg group. AERCAP exhibited non-linear does-effect relationship in this experiment. The non-linear does-effect pattern can be observed in some chemical agents. But it’s more common in crude extract preparation. For instance, morphine stimulates contractions of gut muscle at high dose and inhibits contractions at lower does [26]. The methanol stem-bark extract of *Annona senegalensis Pers.* had anti-diarrheal activity at the dose of 10 mg/kg, but not at the higher dose of 100 mg/kg [27]. Bhuiyan M found that 10 μl of Chan Su extract induced the maximum production of NO on the trophoblastic cell. Then NO production declined with higher quantity of extract [28]. The mechanism might be due to the intricate components of the crude extract, which can act on miscellaneous targets and cause diversity effects. The results we got in the experiments were the integration of various effects. The exact mechanism need to be further explored by new methods such as neural networks based on entropy theory, two-model fuzzy theory *etc.* [29]. 

In the intestinal motility test, we investigated the effect of AERCAP on normal and castor oil stimulated intestinal transit. The positive control drug loperamide exhibited significant inhibition of transit movement in both experiments. Loperamide is an opioid receptor agonist that can act on the μ-opioid receptors in the myenteric plexus to decrease the motility of the circular and longitudinal smooth muscles in the intestinal wall [30]. In AERCAP groups, all doses of AERCAP did not show inhibit effect in normal intestinal transit. It seems that the results are not consistent with some previous reports. The previous reports suggest that crude extract of *Rubia cordifolia* show anticonvulsant activities in many kinds of pharmacologic models in vitro [31]. Several explanations for our conflicting results are possible. First, it is generally believed that the pharmacologic model in vitro can only represent part of the pharmacologic activities in vivo. Second, the contraction activity of intestinal smooth muscle are not completely equal to the longitudinal propulsive movement of intestinal transit [32]. In addition, in the castor oil-induced intestinal transit test, AERCAP inhibited intestinal transit in a dose-dependent manner. Castor oil is also an effective laxative. Its active component, ricinoleic acid, can stimulate electrolyte secretion and promote intraluminal fluid accumulation, which subsequently increases intestinal motility [33]. Thus, based on the results of our study, we hypothesize that the anti- motility effect of AERCAP might be partly mediated by anti-secretion activity, followed by reduced fluid accumulation in the intestinal lumen.

Inflammatory bowel disease (IBD) is a group of inflammatory conditions of the colon and small intestine; however, its etiopathology remains unknown. Diarrhea is a prevalent symptom of IBD. In most cases, it is also the first perceived manifestation that causes the patient to seek medical attention [34]. It has been hypothesized that oxidative stress, immunological abnormal, gut microflora and inflammatory cytokine production might play a role in the initiation and progression of IBD [35]. MDA is a byproduct of lipid peroxidation and is widely used as marker of oxidative stress [36]. MPO is an enzyme that is found in primary granules of polymorphonuclear neutrophils and has been used as a marker of cell-specific infiltration and the severity of inflammation [37]. TNF-α elicits inflammation and cell death and plays an irreplaceable role in the tissue injury [38]. IL-1β is another pro-inflammatory cytokine that promotes immunity and causes local neutrophil recruitment. Analyses of these parameters provided information regarding the probable mechanisms of AERCAP’s protective action in colitis development.

TNBS-induced rat colitis is a well-established animal model of intestinal inflammation, and its clinical and biochemical features resemble those of human IBD [39]. A large amount of evidence indicates that animals subjected to TNBS-induced colitis suffer from diarrhea, weight loss, disorganization of the colon mucosa and sub-mucosa, and significantly increased MDA content, MPO activity, TNF-α and IL-1β [40, 41]. In addition, some plant-derived crude extracts exert anti-inflammatory action by inhibiting lipid peroxidation, oxidant enzyme and inflammatory cytokine production [42, 43].

In our study, rats in the TNBS control group exhibited severe mucosal damage based on the macroscopic and microscopic analyses. The oxidative stress and inflammatory parameters were significantly increased compared with the healthy group. In the AERCAP-treated group, colonic lesions and histological signs of damage were ameliorated. This result indicates the holistic protective action of AERCAP on colitis injury. Further biochemical parameters detection revealed that the MDA content was significantly decreased in the 500 mg/kg AERCAP group compared with the TNBS control group, but no difference in MPO activity was found between the treated colitis group and the TNBS control group. This finding might be due to the extensive damage that sustained the high MPO levels. The TNF-α and IL-1β levels exhibited a more significant decline compared with the TNBS group. These findings indicated the anti-oxidative and anti-inflammatory activities of AERCAP. Meanwhile, previous studies have confirmed that oxidative stress promotes the formation of lipid peroxidation and plays a major role in the pathogenesis of diarrhea [44, 45]. Thus, this finding validated the anti-diarrheal property of AERCAP from an additional aspect.

The qualitative phytochemical screening tests demonstrated that AERCAP are composed primarily of anthraquinones, phenolic acids, tannins and amino acids. The anti-diarrheal and anti-inflammatory effects of AERCAP might be due to the phenolic acid and tannins compounds, since these compounds have been reported previously to possess anti-diarrheal and anti-inflammatory activities [46, 47].

## Conclusions

In summary, our study provided evidence regarding the anti-diarrheal and anti-inflammatory effects of AERCAP. The overall findings validate, at least in part, the indigenous use of AERCAP in the treatment of diarrhea. However, further studies should be performed to continue elucidating the underlying mechanisms to produce a promising alternative anti-diarrhea remedy, and the active component(s) in the crude extract should be isolated and identified.

## Abbreviations

AERCAP: The aqueous extract of *Rubia cordifolia’s* aerial part
EI: Evacuation index
ELISA: Enzyme-linked immunosorbent assay
H_2_O_2_: Hydrogen peroxide
IBD: Inflammatory bowel disease
MDA: Malondialdehyde
MPO: Myeloperoxidase
TBA: Thiobarbituric acid
TNBS: Trinitrobenzenesulfonic acid
